# Mechanism for the lethal effect of enterovirus A71 intracerebral injection in neonatal mice

**DOI:** 10.1038/s41374-019-0351-5

**Published:** 2019-12-19

**Authors:** Min Feng, Yun Liao, Yang Gao, Guorun Jiang, Lichun Wang, Ying Zhang, Shengtao Fan, Xingli Xu, Qihan Li

**Affiliations:** Yunnan Key Laboratory of Vaccine Research and Development on Severe Infections Diseases, Institute of Medical Biology, Chinese Academy of Medicine Sciences and Peking Union Medical College, Kunming, 650118 China

**Keywords:** Viral infection, Infection

## Abstract

Enterovirus A71 (EV-A71) infection is primarily responsible for fatal hand, foot, and mouth disease (HFMD) cases. Infants and younger children are more likely to suffer central nervous system damage as a result of EV-A71 infection, but this virus mostly does not affect older children and adults. This study investigated the possible mechanism underlying the age-dependent lethal effect of EV-A71 infection by comparing neonatal and adult mouse models of EV-A71 infection. Although viral proliferation is absent in both neonatal and adult mice, we observed that EV-A71, as a stimulus for astrocytes, elevates the levels of cytokines and monoamine neurotransmitters in neonatal mice. Then, we selected IL-6 and adrenaline as targets in a pharmacological approach to further validate the roles of these factors in mediating the mortality of neonatal mice after EV-A71 infection. Intracerebral injection of IL-6 and adrenaline enhanced the severity of EV-A71 infection, while treatment with an anti-IL-6-neutralizing antibody or the adrenergic-antagonist phenoxybenzamine reversed the lethal effect of EV-A71 in neonatal mice. These results suggest that the central nervous system (CNS) damage in neonatal cases of EV-A71 infection might be caused by an activated fetal cerebral immune response to the virus, including the disruption of brainstem function through increased levels of cytokines and neurotransmitters, rather than the typical cytopathic effect (CPE) of viral infection.

## Abstract (web summary)

The central nervous system damage in neonatal mice with EV-A71 infection may be caused by activated fetal cerebral astrocytes related to the immune response to the virus, including the disruption of the functioning of the brainstem through increased cytokines and neurotransmitters, rather than the typical cytopathic effect of viral infection.

## Introduction

Since 1997, hand, foot, and mouth disease (HFMD) has been an infectious disease that seriously affects the health of children in East and Southeast Asia [1, 2]. In several large outbreaks, HFMD associated with enterovirus A71 (EV-A71) has appeared as the primary threat in a few patients who developed severe complications or even experienced death [3]. Clinical observations reveal that infants and younger children are more likely to exhibit central nervous system (CNS) damage as a result of EV-A71 infection, while this virus generally does not affect older children and adults [4]. Therefore, the following question should be posed: what is the mechanism that underlies this age-dependent susceptibility to EV-A71 infection? Various animal models have been used to study EV-A71 pathogenesis. From the perspective of ethical and economic considerations, neonatal mice are reliable models that mimic the severe neurotropic infection of EV-A71 [5, 6]. Moreover, mouse models exhibit an age-dependent effect similar to that observed in humans, as mice older than 2 weeks of age are usually resistant to non-mouse-adapted EV-A71 strains [7]. Consequently, determining the mechanism that underlies the age-dependent lethal effect of EV-A71 in mice may provide clues for better understanding the pathogenesis of fatal complications in infants and younger children.

In our previous work, by using a rhesus macaque infection model and human autopsy, we found that EV-A71 can enter the CNS and generate a severe inflammatory response [8]. Astrocytes may play a major role in the pathogenesis of EV-A71 infection [8, 9]. Except for viruses that directly infect and proliferate in astrocytes, viral infections most likely occur through the capacity of astrocytes to modify neurological functions by serving as active immune cells with modulatory neuronal functions [10, 11]. For example, the upregulation of cytokines from infected astrocytes may stimulate neurons and increase neurotransmitter release. Under certain conditions, this antiviral immune response may become an active contributor to CNS damage and a series of neurogenic clinical symptoms related to the high rate of mortality. Combined with our pilot work, neonatal mice usually died within 5–7 days postintracerebral EV-A71 injection, while adult mice did not show any clinical symptoms. We hypothesized that increased levels of cytokines and neurotransmitters may play a critical role in the lethal effect of EV-A71 infection in neonatal mice.

Herein, we first duplicated the dynamic progression of EV-A71 infection in the CNS of neonatal pups and adult mice. In addition, histopathological examinations were performed to determine whether astrocytes are involved in EV-A71 infection in neonatal mice. Then, we cultured murine astrocytes and described the viral growth curve and biological characteristics in infected cell cultures. In vivo investigations with the mouse model were performed to confirm that EV-A71-infected astrocytes were associated with the increased expression of proinflammatory cytokines and adrenaline in the brain. In addition, we chose IL-6 and adrenaline as targets in a pharmacological approach to further validate the roles of these factors in mediating the mortality of neonatal mice subjected to EV-A71 infection. This study collected data through in vitro and in vivo systems and compared neonatal mice with adults. If EV-A71 infection induces astrocyte dysfunction and the expression of cytokines and monoamine neurotransmitters is associated with its lethal effect, then this situation could be reversed by anti-IL-6-neutralizing antibodies and adrenergic antagonists.

## Materials and methods

### Virus and cell culture

The EV-A71 virus strains FY-22 (GenBank: EU913466) and FY-23 (GenBank: EU812515) were used in this study. These strains belong to subgenogroup C4. FY-22 and FY-23 were isolated from the respiratory tract secretions of a patient with a mild EV-A71 infection and a child with severe cardiopulmonary collapse, respectively, in Fuyang, China, in May 2008 [12]. The virus was expanded using Vero cells (American Type Culture Collection, USA) and harvested following the development of a typical cytopathic effect (CPE). Vero cells were maintained in Dulbecco’s modified Eagle’s medium (DMEM; Corning, USA) supplemented with 10% fetal bovine serum (FBS) (Gibco, USA), 100 U/ml penicillin, and 100 μg/ml streptomycin at 37 °C with 5% CO_2_. The culture medium was changed to DMEM supplemented with 2% FBS after viral infection. The EV-A71 strains were also titered on Vero cells.

### Virus titration

Virus titration was performed using a microtitration assay according to a standard protocol [13]. Briefly, viral stocks were subjected to serial tenfold dilutions, added to 96-well plates coated with Vero cells and incubated at 37 °C in 5% CO_2_. The presence of a CPE was recorded at 7 days post infection (dpi).

### Animals

Specific-pathogen-free imprinting control region mice (pregnant mice and 6-week-old female mice) were obtained from the Department of Small Animal of the Institute of Medical Biology, Chinese Academy of Medicine Sciences. The mice were singly housed in polycarbonate cages under 12 h light/dark conditions (lights on between 8:00 a.m. and 8:00 p.m.). Neonatal mice remained with the dams during the experimental phase. The room temperature was maintained at 22 ± 2 °C, and food and water were available ad libitum. All experimental procedures were reviewed and approved by the Yunnan Provincial Experimental Animal Management Association (approval number: SCXK (Dian) 2011–0005) and the Experimental Animal Ethics Committee of the institute (approval number: YIKESHENGLUNZI [2016] 54).

### Astrocyte separation and culture

Astrocytes were separated from the cortex, mid brain, pons, and medulla oblongata of mice and cultured as previously described [14]. Briefly, the brain tissues of sacrificed animals were harvested, washed several times with PBS, sheared, digested with trypsin, filtered and cultured in DMEM containing 10% FBS and antibiotics at 37 °C in 5% CO_2_. The primary cell culture was grown to confluence and washed three times with 0.01 M PBS before the addition of 0.0625% trypsin. The digested cells were centrifuged and suspended for fibroblast removal by differential adhesion for 30 min. The suspension was transferred to another culture plate for adhesion for 60 min, followed by the removal of microglia and oligodendroglia to obtain purified astrocytes, which were identified with green fluorescent anti-glial fibrillary acidic protein (GFAP) antibody following three rounds of purification.

### EV-A71-infected astrocytes in vitro

Astrocytes were infected with EV-A71 (multiplicity of infection = 0.1) for 60 min at 37 °C and washed twice with PBS. Then, 2% FBS was added prior to continuous incubation. The cell culture supernatants were harvested at different time points and stored at −80 °C prior to further analyses.

### Extraction of viral RNA, PCR, and quantitative real-time PCR (qRT-PCR)

Viral RNA was extracted from EV-A71-infected astrocytes or Vero cells using the MiniBEST Viral RNA/DNA Extraction Kit (Takara, Japan) according to the manufacturer’s instructions. The presence of EV-A71 negative-strand RNA was detected using a PrimeScript RT reagent Kit (Takara, Japan) on a Mastercycler nexus GSX1 thermal cycler (Eppendorf, Germany), and qRT-PCR amplification was performed using Taqman 1-step RT-PCR Master Mix (Takara, Japan) on a 7500 Fast Real-time RT-PCR system (Applied Biosystems, USA), as previously described [15, 16]. The primers for *RPL13A* (internal control) were 5′-CCTTGGAGGAGAAGAGGAAAGAGA-3′ and 5′-TTGAGGACCTCTGTGTATTTGTCAA-3′.

### Cytokine and monoamine analyses

The cytokine levels in the culture supernatants and homogenized brain samples were evaluated using ELISA kits (Neobioscience Technology Co. Ltd, China). Adrenaline levels were analyzed with a 3-CAT Research EIA Kit (Demeditec Diagnostics GmbH, Germany). These tests were carried out according to the manufacturer’s instructions. A sample volume of 100 μl was used to determine the cytokine levels in the culture supernatant. For the determination of cytokines in brain homogenates, the brain tissue was weighed, and an appropriate amount of lysis buffer was added to achieve 100 mg tissue/75 μl buffer; then, the sample was ground, homogenized, and centrifuged, and the supernatants were transferred. Next, 100 μl of 20–50-fold diluted supernatants were added to ELISA plates. For the determination of adrenaline in the brain homogenates, 800 μl PBS (with 1 mM EDTA) was added to a weighed sample of brain tissue; the sample was ground, homogenized, and centrifuged. The supernatants were then transferred, and the sample size was 200 μl for the subsequent assay.

### Infection of neonatal mice

All neonatal mice (within 48 h of birth) were intracranially injected at the midpoint between the outer edge of the eye and the leading edge of the external ear, and adult mice (6 weeks old) were intracranially injected at an oblique orientation above the canthus [17]. The injection volume was 20 μl using 0.25-ml sterile syringes (needle, 4.5; diameter, 0.45 mm).

To detect the virus loads in the mouse brain, neonatal or adult mice were intracerebrally injected with EV-A71 (10^4.5^ TCID_50_/animal) and subsequently sacrificed at different time points post injection. In the infection experiment, neonatal mice were randomly divided into EV-A71-infected, inactivated virus, and control groups. The mice in the infected group were injected with EV-A71 (10^4.5^ TCID_50_/animal), the mice in the inactivated virus group were injected with inactivated virus (containing the same quantity of viral antigen as the live virus group), and the mice in the control group were injected with the same volume of PBS. The mice were sacrificed at 4 dpi, and the brains were removed for subsequent pathological examination.

For the quantification of mouse survival, neonatal mice were randomly divided into different groups and treated with IL-6 (1 ng/mouse, ic), adrenaline (1 μg/mouse, ic), an anti-IL-6-neutralizing antibody (20 ng/mouse, ic), phenoxybenzamine (α-adrenergic-antagonists, 0.5 μg/mouse, sc), or PBS. Ten minutes later, all mice were infected with a strongly virulent strain (FY-23), a weakly virulent strain (FY-22) of EV-A71 (10^4^ CCID_50_ per mouse), or PBS. The survival of the mice was recorded over 7 dpi (for the FY-23 groups) or 10 dpi (for the FY-22 groups).

The number of animals used per group is shown in Table 1.

**Table 1:** A schematic depiction of the number of animals used in each experimental group.

Experiment	Age of animals	Treatment and sacrificed time point		Number of animals	Note
Astrocyte separation and culture	Neonatal	Within 24 h of birth		42	
HE, IHC, and IHF staining	Neonatal	Virus	4 dpi	4	
		PBS		4	
Cytokine and monoamine detection	Neonatal	Virus	4 dpi	11	Half brain for cytokine, and half for monoamine test
		Inactivated Virus		7	
		PBS		13	
Cytokine and monoamine detection	Adult	Virus	4 dpi	4	
		PBS		6	
Viral load	Neonatal	Virus	2 hpi	3	
			24 hpi	4	
			48 hpi	4	
			72 hpi	4	
			96 hpi	3	
			120 hpi	3	
	Adult		2 hpi	4	
			24 hpi	4	
			48 hpi	4	
			72 hpi	3	
			96 hpi	3	
			120 hpi	3	
Survival	Neonatal	PBS		11	Figure 4a
		FY-22		16	
		IL-6		10	
		FY-22 + IL-6		14	
		PBS		10	Figure 4b
		FY-22		16	
		AD		12	
		FY-22 + AD		16	
		PBS		7	Figure 4c
		FY-23		10	
		FY-23 + IL-6Ab		9	
		PBS		8	Figure 4d
		FY-23		11	
		FY-23 + Phenoxybenzamine		10	

*dpi* days post infection, *hpi* hours post infection, *AD* adrenaline

### Histopathological, immunofluorescence, and immunohistochemical assays

Brain samples from the experimental animals were fixed in 10% formalin in PBS, dehydrated in graded ethanol, and embedded in paraffin before obtaining 4-µm sections for further experiments, including hematoxylin and eosin staining, immunofluorescence assays, and immunohistochemical assays. The EV-A71 antigen was detected using a primary mouse anti-EV-A71 monoclonal antibody (Chemicon, USA) and a secondary horseradish peroxidase (HRP)-conjugated anti-mouse IgG antibody (Sigma, Germany) in immunohistochemical analyses or an Alexa Fluor 594-conjugated donkey anti-mouse IgG antibody (Life Technologies, USA) in immunofluorescence assays. Astrocytes were detected using a rabbit anti-GFAP antibody (Abcam Ltd., UK) as the primary antibody and an Alexa Fluor 488 conjugated donkey anti-rabbit IgG antibody (Life Technologies, USA) as the secondary antibody. The staining procedure was performed according to a standard protocol [15, 18]. The histopathological and immunohistochemical analyses were performed using a light microscope (Nikon DS-Ril/Eclipse), and the immunofluorescence assay was performed with a Leica SP8 laser scanning confocal microscope system.

### Statistical analysis

All data except survival rates are expressed as the mean + SD (or SEM), and the differences between the two groups were evaluated using independent samples *t-*tests. The mouse survival data were quantified and analyzed by the log-rank test. For all comparisons, a significant difference was assumed at *p* ≤ 0.05; all of the data were analyzed using SPSS version 21 or GraphPad Prism 7.00.

## Results

### Astrocytes were involved in the lethal effect of EV-A71 intracerebral injection in neonatal mice

**Fig. 1:**
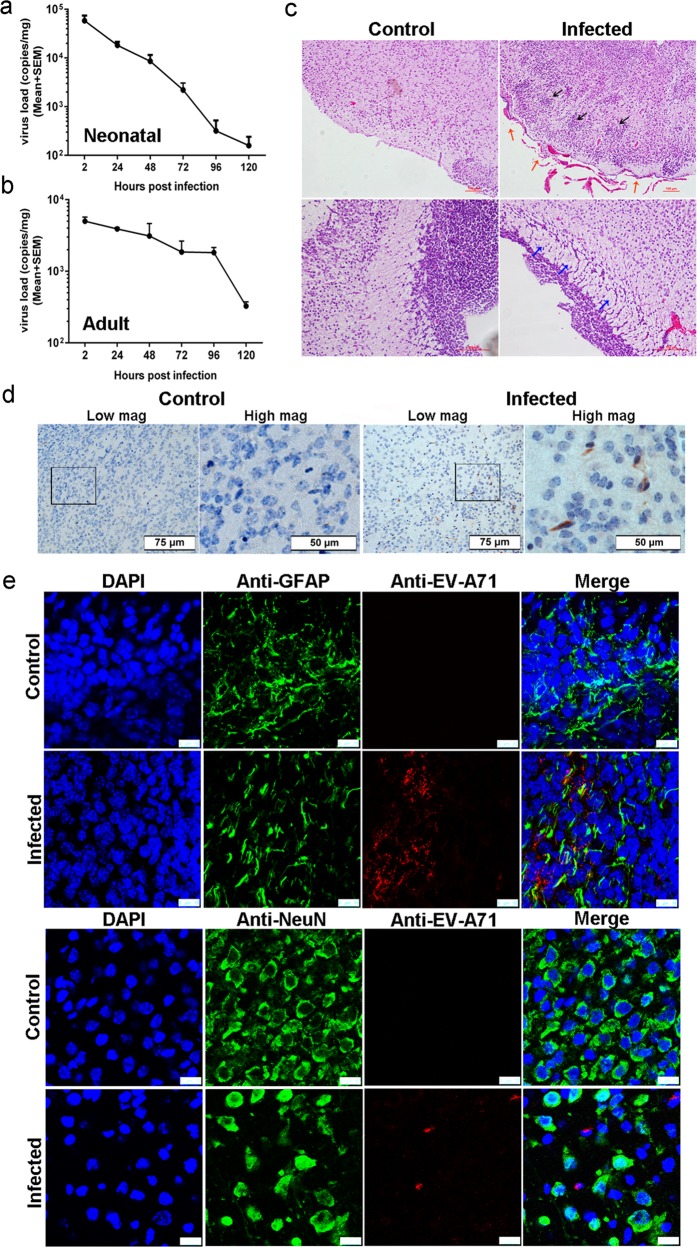
Astrocytes were involved in the lethal effect of EV-A71 intracerebral injection in neonatal mice. Proliferation of the virus in neonatal (**a**) and adult (**b**) mice brain. **c** Representative HE staining of pathological lesions in brainstem, including glial cell proliferation and glial nodule formation (black arrows), vascular congestion and lymphocyte infiltration (red arrows), and swollen and loose arrangement cells (blue arrows). **d** Viral antigen expression in the brainstem tissues from EV-A71-infected neonatal mice, scale bars are shown at bottom right. **e** Immunofluorescence confocal microscopy observations of astrocytes (or neurons) and EV-A71 antigen in brainstem tissues from infected neonatal mice, scale bar = 10 μm.

In our pilot work and previous studies [8, 19], the intracerebral injection of EV-A71 caused death in neonatal mice but not in adult mice after 5–7 days. The infected neonatal mice had list of clinical symptoms, such as weight loss, directional movement disorders, hindlimb paralysis, and even death [19]. We speculated that the virus may proliferate in neonatal mice and induce this lethal effect. Thus, we first evaluated the viral kinetic growth curve after intracerebral injection of EV-A71. Unexpectedly, the results revealed that the virus decreased during the 5 dpi in both neonatal (Fig. 1a) and adult (Fig. 1b) mouse brains. To investigate the cause of death in neonatal mice without virus proliferation, we performed pathological examinations and tracked the virus in the neonatal mouse brains. The histopathological manifestations in the CNS were characterized as glial cell proliferation and glial nodule formation, vascular congestion and lymphocytes infiltrates in the meninges, and partial cells located near ventricles that were swollen with a loose arrangement (Fig. 1c), and viral antigen was detected in the brainstem (Fig. 1d). Considering the preferential infection of astrocytes by EV-A71 revealed in our previous study using neonatal rhesus macaques and human autopsy specimens [8], we performed an immunofluorescence confocal microscopic analysis of the viral antigen and astrocytes in the CNS tissues collected from the EV-A71-infected neonatal mice. The presence of EV-A71 in astrocytes was confirmed based on coimmunolabeling with the red fluorescent anti-EV-A71 antibody and the green fluorescent anti-GFAP antibody (an astrocyte marker) (Fig. 1e). Observation of the brainstems showed that 22.58% of the astrocytes from a total of 100 random fields from 20 sections taken from four infected neonatal mice at 4 dpi were antigen positive. These findings suggested that EV-A71 could enter astrocytes but barely replicate, indicating a potential role of astrocytes in neonatal mice with EV-A71 infection.

### Modulation of cytokine release from astrocytes stimulated by virus in vitro

**Fig. 2:**
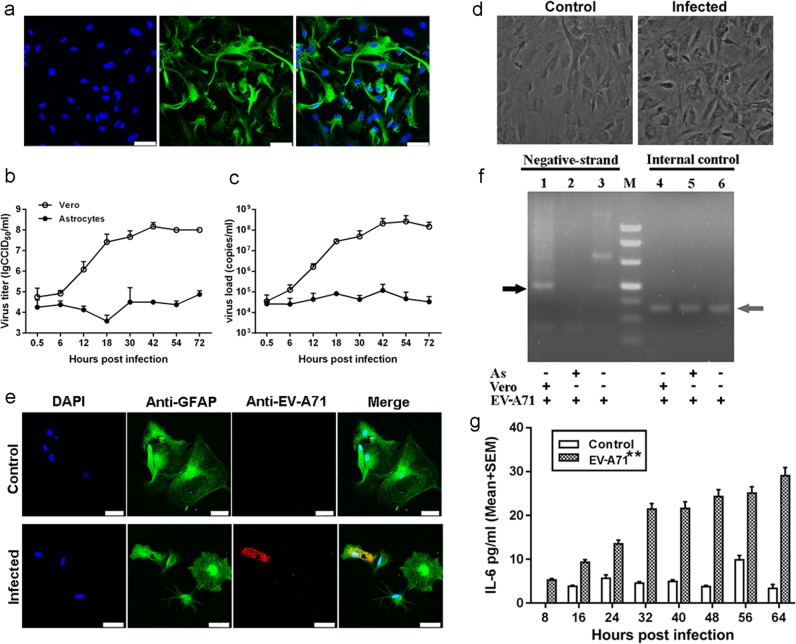
The modulation of cytokine release from astrocytes stimulated by virus in vitro. **a** The astrocytes from neonatal mice were grown on glass slides and stained with an anti-GFAP antibody; scale bar = 50 μm. Proliferations of the virus in cultured mouse astrocytes (**b**) and Vero cells (**c**) were measured based on virus titration from 0.5 to 72 h post infection. **d** Microscopy observations of EV-A71-infected cultured mice astrocytes, images are shown at ×400 magnification. **e** Immunofluorescence confocal microscopy observations of astrocytes and EV-A71 antigen in cultured mouse astrocytes; scale bar = 50 μm. **f** Detection of the presence of EV-A71 negative-stranded RNA in cells (black arrow). M marker; AS astrocyte; Vero Vero cells. **g** IL-6 released by astrocytes from 6 to 64 h post infection. **p ≤ 0.01 compared with the corresponding control group.

To further detect the involvement of astrocytes in EV-A71 infection of the CNS, astrocytes were separated from the brains of mice and cultured. Cells from the third passage were identified based on a specific marker, GFAP, using a fluorescent antibody, and the results showed that more than 95% of these cells were astrocytes (Fig. 2a). The EV-A71 growth dynamic profile in the cultured cells revealed limited viral proliferative activity in murine astrocytes (Fig. 2b, c), although some cells displayed slight swelling and rounding (Fig. 2d). In addition, the detection of immunolabeling using a red fluorescent anti-EV-A71 antibody indicated a certain degree of positive results in astrocytes 48 h post infection (hpi) (Fig. 2e). In addition, 18.6% of the astrocytes counted from 30 random fields from three slides of infected astrocytic culture were antigen positive. To determine whether EV-A71 replicates in murine astrocytes, we attempted to detect the presence of EV-A71 negative-strand RNA in astrocyte cultures. In parallel, Vero cells that are sensitive to EV-A71 infection and the virus itself were used as positive and negative controls, respectively. However, this result may reveal a lack of viral replication in murine astrocytes (Fig. 2f). Collectively, these findings suggest that EV-A71 could be present in astrocytes and that the viruses may not proliferate, implying that EV-A71 may have an unidentified route of entry into murine astrocytes, even in the absence of replication. To understand the pathophysiological characteristics of the infection of astrocytes with EV-A71, we measured the levels of different proinflammatory cytokines in the supernatant of virus-infected astrocytes from mice. The results indicated that there was a marked upregulation of IL-6 and IL-8 levels (*ps* < 0.05) within 64 hpi in astrocytes from mice (Fig. 2h and Fig. S1a), although the virus did not show obvious replication in astrocytes. This finding suggests that the EV-A71 antigen may be a stimulus that affects the function of astrocytes in vitro.

### Upregulated cytokines and neurotransmitters in the brains of infected neonatal mice compared with adult mice in vivo

**Fig. 3:**
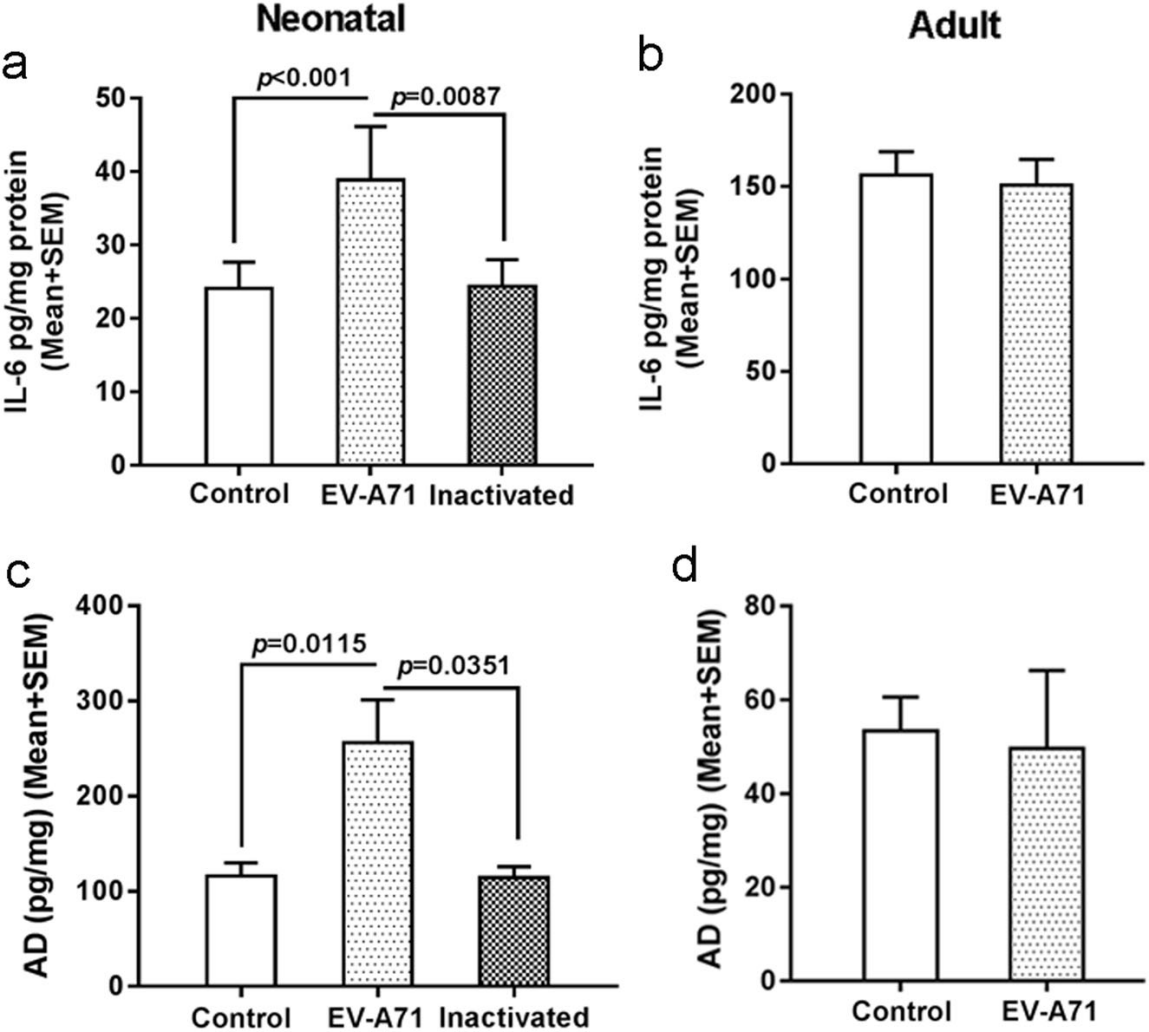
Upregulated cytokines and neurotransmitters in the brains of infected neonatal mice compared with the brains of adult mice in vivo. IL-6 (**a**, **b**) and adrenaline (**c**, **d**) detection in brainstem homogenates from EV-A71 infected neonatal and adult mice. AD adrenaline.

Based on the detection of EV-A71-infected astrocytes in vitro, it is critical to examine the variation in cytokine production and release in vivo. As expected, elevated levels of multiple proinflammatory cytokines, including IL-6 and IL-8 (*ps* < 0.05), were found in the brain homogenates of neonatal mice treated with live EV-A71 (Fig. 3a and Fig. S1b), and these results are largely in agreement with previous observations in vitro. Next, based on the structural homogeneity of IL-6 in rhesus monkeys and humans [20], the elevated levels of IL-6 released from infected astrocytes occurred concomitantly with increased adrenaline release in the CNS. Consistently, examination of homogenates from the neonatal mouse brains injected intracerebrally with live virus revealed an increased level of adrenaline (*p* < 0.05) (Fig. 3b). In comparison, the levels of proinflammatory cytokines (Fig. 3c and Fig. S1c) and adrenaline (Fig. 3d) did not show obvious changes in inactivated virus-treated neonatal mouse brains or live virus-treated adult mouse brains.

### IL-6 and adrenaline mediated the lethal effect in neonatal mice injected intracerebrally with EV-A71

**Fig. 4:**
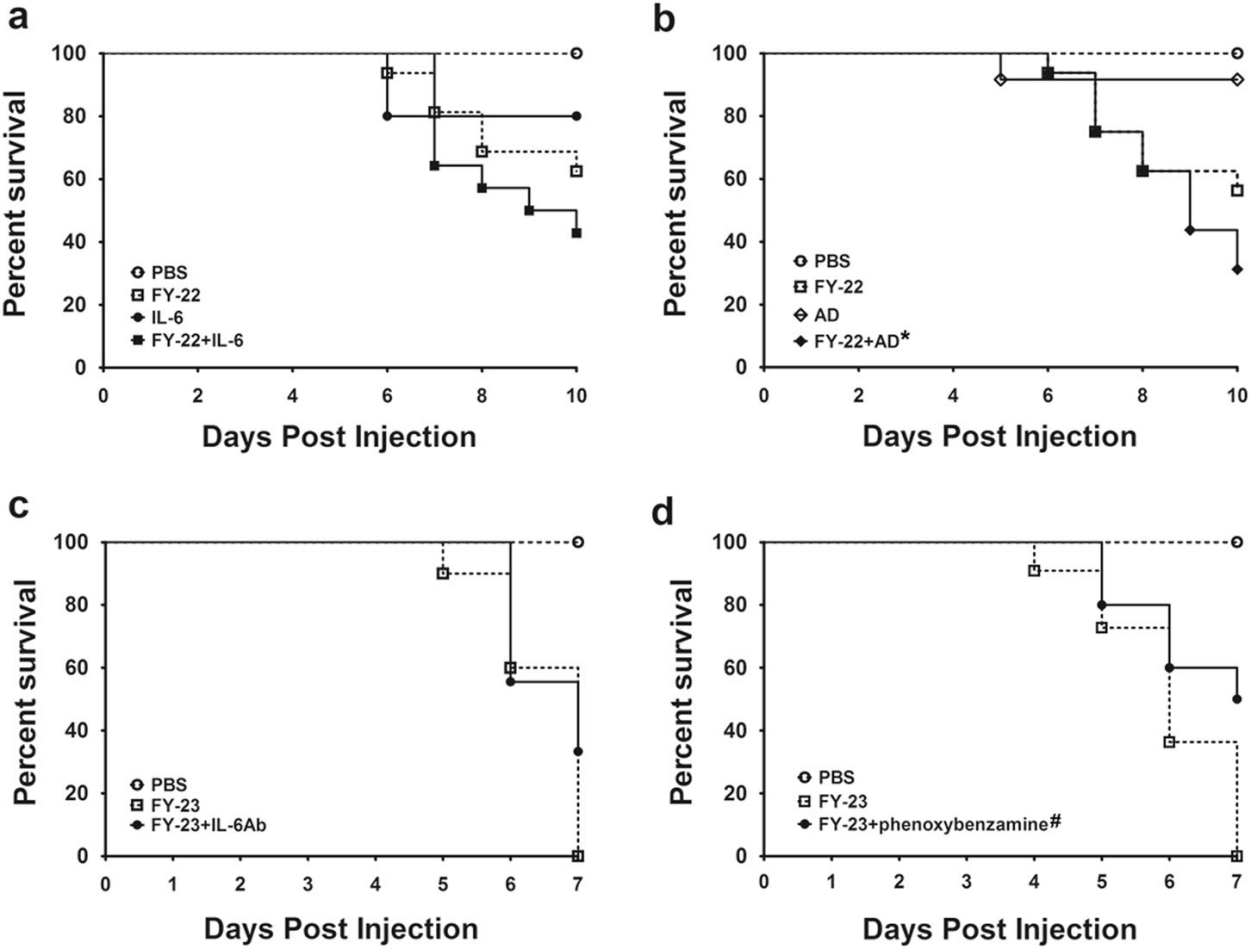
IL-6 and adrenaline mediated the lethal effect in neonatal mice injected intracerebrally with EV-A71. **a** IL-6 (1 ng/mouse, ic) and **b** adrenaline (1 μg/mouse, ic) decreased the survival rate of neonatal mice infected with FY-22. **c** An anti-IL-6 neutralizing antibody (20 ng/mouse, ic), and **d** the adrenergic-antagonist phenoxybenzamine (500 ng/mouse, sc) increased the survival rate of EV-A71-infected suckling mice. AD adrenaline. *p < 0.05 in comparison to the AD group; ^#^*p* < 0.05 in comparison to the corresponding FY-23 group.

To further verify whether IL-6 and adrenaline mediate the lethal effect on EV71-infected neonatal mice, two EV-A71 strains were used in lethal challenge. One EV-A71 strain is a weaker viral strain (FY-22) that causes death in some mice (37.50–43.75%) within 10 dpi (Fig. 4a, b), while another EV-A71 strain is a highly virulent strain (FY-23) with a 100% death rate within 7 dpi (Fig. 4c, d). First, the FY-22 combined with IL-6 or adrenaline groups were compared with their respective FY-22-only groups. The death rates of the coinjection groups were increased (57.13% for the FY-22 + IL-6 group, 68.75% for the FY-22 + adrenaline group) (Fig. 4a, b), confirming that enhanced IL-6 and adrenaline can aggravate the situation during EV-A71 infection. Second, to examine the reversing effect of the anti-IL-6-neutralizing antibody and the adrenergic antagonist, these two drugs were administered with the virulent FY-23 strain. In contrast to that in the FY-23-only groups, the death rate in the group treated with anti-IL-6-neutralizing antibody was reduced by 33.33% (Fig. 4c). The adrenergic antagonist also induced a substantial 50% reduction in the death rate (*p* < 0.05, log-rank test) (Fig. 4d). Together, IL-6 and adrenaline increased the death rate of neonatal mice during EV-A71 infection. Conversely, anti-IL-6-neutralizing antibody and the adrenergic antagonist decreased the death rate. These two findings indicated that elevated IL-6 and adrenaline play key roles in inducing death during EV-A71 infection in neonatal mice.

## Discussion

This study reveals the potential mechanisms responsible for the lethal effect of EV-A71 intracerebral injection in neonatal mice. As we report in the present study, neonatal mice died within 7 dpi, but there is no viral proliferation. This lethal effect of EV-A71 injection in neonatal mice is partly due to astrocyte dysfunction, following the increased expression of cytokines, the stimulation of neurons by these upregulated cytokines may lead to a disturbed homeostasis of monoamine neurotransmitters [8]. Astrocytes, the most abundant glial cells in the CNS, are important for the maintenance of CNS homeostasis, and these cells can modulate neuronal functions based on specific physiological capabilities [21], such as their ability to engulf invading pathogens and release active factors. In vitro, EV-A71 can enter mouse astrocytes, but in the absence of viral replication, this process is accompanied by increased IL-6 and IL-8 levels in the supernatant. In vivo, the analysis of homogenized brainstem tissue also demonstrated the upregulation of cytokines, including IL-6 and IL-8, and adrenaline, in infected neonatal mice, but not in the inactivated antigen-treated group. These findings are consistent with our previous findings [8] and those of other studies [22–24].

In our previous study [8], to investigate the potential interaction of astrocytes and neurons during EV-A71 infection, we designed a double-layer coculture system consisting of an upper layer of EV-A71-infected human astrocytes and a lower layer of uninfected human neurons from the brainstem. Detection of neurotransmitters in the supernatant of the neuron layer revealed that adrenaline increased significantly after 36 h after exposure to the infected astrocyte layer. To confirm this increase in adrenaline, we directly added IL-6 or IL-8 to cultured neurons, and the results indicated that IL-6 induced a significant upregulation of adrenaline, whereas IL-8 had no effect. Thus, we chose IL-6 and adrenaline as targets of the pharmacological approach to further validate their roles in mediating the mortality of neonatal mice with EV-A71 infection, although IL-8 was also upregulated in infected astrocytes and brain tissue. The results of the pharmacological tests provided direct evidence that intracerebral injection of IL-6 and adrenaline enhanced the severity of EV-A71 infection, while injection of an anti-IL-6-neutralizing antibody and the adrenergic antagonist phenoxybenzamine reversed the lethal effect of EV-A71 in neonatal mice. However, although both neonatal and adult mice received identical EV-A71 intracerebral injections, adult mice showed neither increased expression of cytokines or neurotransmitters nor neurological symptoms or death. The distinct age-dependent lethal effect reflects a differential host intracerebral immune response to live virus. Neonatal individuals and infants may have greater difficulty maintaining neural homeostasis than adults [25]. This effect may be related to an immature nervous system that cannot quickly detect potentially deleterious changes in neural state or make appropriate corrective responses. In the absence of viral proliferation, CNS damage in neonatal cases with EV-A71 infection is caused by an activated fetal cerebral immune response to the virus, including increased cytokines and neurotransmitters that disrupt the function of the brainstem, rather than by the typical CPE of viral infection.

A fundamental issue related to EV-A71 infection of the CNS is the naturally targeted cells in the CNS. Considering the effects of poliovirus [26], which can replicate in and destroy motor neurons, most research on EV-A71 infection has focused on neurons, showing that viral antigens are expressed on neurons and that neuronal damage plays a critical role in EV-A71 encephalomyelitis [27]. However, knowledge about the other host cells of EV-A71 in the CNS is limited. With growing research in this field, observations have shown that other cells in the CNS, including astrocytes [8, 9, 28], microglia [29, 30], etc., may also be involved in EV71 infection. For example, our previous study showed that 0.25 and 0.32% of neurons and 91 and 89% of astrocytes were antigen positive in macaques and human patients, respectively [8]. These findings are consistent with other studies [9] that have shown that viral replication occurs in some neurons but is mostly limited to astrocytes for an unknown reason. In this study, double immunofluorescence staining for viral antigens with markers of astrocytes or neurons also suggested a preference for astrocytes over neurons in neonatal mice. Although the exact mechanism behind the preference of EV-A71 for astrocytes has not yet been elucidated, our previous and present work offers a new perspective and will help elucidate the pathogenesis of EV-A71 infection in the CNS. Future work should systematically investigate the host cells of enteroviruses in the CNS and the consequences of disease progression.

We observed that EV-A71, as an astrocyte stimulus, elevates the level of cytokines; then, the increased cytokines stimulate neuronal secretion of the monoamine neurotransmitter adrenaline [8]. The disruption of brainstem homeostasis might play a leading role in the pathophysiological mechanism of the EV-A71-induced lethal effect. In this context, the lethal effect on neonatal mice may not be due to the unique characteristics of EV-A71 infection. Human enteroviruses, including poliovirus, coxsackievirus, and echovirus, induce death in neonatal mouse models, which may also be related to this pathophysiological mechanism. We propose that early therapeutic intervention, for example, treatment with anti-IL-6-neutralizing antibodies and adrenergic antagonists, may preempt the robust proinflammatory response and boost prorepair mechanisms to maintain neural homeostasis. In addition, an interesting finding was the different virus infection dynamic processes among astrocytes of various species. EV-A71 can enter and proliferate in human and macaque astrocytes with obvious CPEs [8], but viruses can be present in mouse astrocytes but without obvious proliferation or CPE. This phenomenon is most likely due to a deficiency of appropriate EV-A71 receptors or the fact that certain cells lack essential factors for efficient EV-A71 translation and replication [31]. Additional experiments are needed in the future to determine the direct mechanisms that underlie this phenomenon, which may be critical to understanding the pathogenesis of EV-A71 infection.

## Supplementary information

Supplemental Figure

## References

[1] Xing W, Liao Q, Viboud C, Zhang J, Sun J, Wu JT (2014). Hand, foot, and mouth disease in China, 2008–12: an epidemiological study. Lancet Infect Dis.

[2] Aswathyraj S, Arunkumar G, Alidjinou EK, Hober D (2016). Hand, foot and mouth disease (HFMD): emerging epidemiology and the need for a vaccine strategy. Med Microbiol Immunol.

[3] Ooi MH, Solomon T, Podin Y, Mohan A, Akin W, Yusuf MA (2007). Evaluation of different clinical sample types in diagnosis of human enterovirus 71-associated hand-foot-and-mouth disease. J Clin Microbiol.

[4] Mao LX, Wu B, Bao WX, Han FA, Xu L, Ge QJ (2010). Epidemiology of hand, foot, and mouth disease and genotype characterization of Enterovirus 71 in Jiangsu, China. J Clin Virol.

[5] Yu P, Bao L, Xu L, Li F, Lv Q, Deng W, et al. Neurotropism in vitro and mouse models of severe and mild infection with clinical strains of enterovirus 71. Viruses. 2017;9:351. 10.3390/v9110351 PMC5707558 29156632

[6] Zhongping X, Hua L, Ting Y, Zhengling L, Min F, Tianhong X (2016). Biological characteristics of different epidemic enterovirus 71 strains and their pathogeneses in neonatal mice and rhesus monkeys. Virus Res.

[7] Wang YF, Yu CK (2014). Animal models of enterovirus 71 infection: applications and limitations. J Biomed Sci.

[8] Feng M, Guo S, Fan S, Zeng X, Zhang Y, Liao Y (2016). The preferential infection of astrocytes by enterovirus 71 plays a key role in the viral neurogenic pathogenesis. Front Cell Infect Microbiol.

[9] Hao B, Gao D, Tang DW, Wang XG, Liu SP, Kong XP (2012). [Distribution of human enterovirus 71 in brainstem of infants with brain stem encephalitis and infection mechanism]. Fa yi xue za zhi.

[10] Haydon PG (2000). Neuroglial networks: neurons and glia talk to each other. Curr Biol.

[11] Sofroniew MV, Vinters HV (2010). Astrocytes: biology and pathology. Acta Neuropathol.

[12] Wang LC, Tang SQ, Li YM, Zhao HL, Dong CH, Cui PF (2010). A comparison of the biological characteristics of EV71 C4 subtypes from different epidemic strains. Virologica Sinica.

[13] Pizzi M (1950). Sampling variation of the fifty percent end-point, determined by the Reed-Muench (Behrens) method. Hum Biol.

[14] Yue L, Guo S, Zhang Y, Liu L, Wang Q, Wang X (2013). The modulation of phosphatase expression impacts the proliferation efficiency of HSV-1 in infected astrocytes. PLoS ONE.

[15] Liu L, Zhao H, Zhang Y, Wang J, Che Y, Dong C (2011). Neonatal rhesus monkey is a potential animal model for studying pathogenesis of EV71 infection. Virology.

[16] Ma N. Study of EV71 infected astrocytes and its pathological significance. Vol. Master. China: Kunming Medical University, 2013.

[17] Li Hui WC, Shan Luo, Hongbing Wang, Xiaowu Huang (2013). Research for the methods of mouse intracephalic injection. Lab Animal Sci.

[18] Jiang Z, Wu CL, Woda BA, Iczkowski KA, Chu PG, Tretiakova MS (2004). Alpha-methylacyl-CoA racemase: a multi-institutional study of a new prostate cancer marker. Histopathology.

[19] Fan S, Wang L, Zhao H, Li W, Li L, Liu L (2011). Interferon protects suckling ICR mice against enterovirus 71 infection. Chin J Biol.

[20] Villinger F, Brar SS, Mayne A, Chikkala N, Ansari AA (1995). Comparative sequence analysis of cytokine genes from human and nonhuman primates. J Immunol.

[21] Mucke L, Eddleston M (1993). Astrocytes in infectious and immune-mediated diseases of the central nervous system. FASEB J.

[22] Liao YT, Wang SM, Wang JR, Yu CK, Liu CC (2015). Norepinephrine and epinephrine enhanced the infectivity of enterovirus 71. PLoS ONE.

[23] Wang SM, Lei HY, Su LY, Wu JM, Yu CK, Wang JR (2007). Cerebrospinal fluid cytokines in enterovirus 71 brain stem encephalitis and echovirus meningitis infections of varying severity. Clin Microbiol Infect.

[24] Khong WX, Foo DG, Trasti SL, Tan EL, Alonso S (2011). Sustained high levels of interleukin-6 contribute to the pathogenesis of enterovirus 71 in a neonate mouse model. J Virol.

[25] Levy O (2007). Innate immunity of the newborn: basic mechanisms and clinical correlates. Nat Rev Immunol.

[26] Nomoto A, Koike S, Aoki J (1994). Tissue tropism and species specificity of poliovirus infection. Trends Microbiol.

[27] Weng KF, Chen LL, Huang PN, Shih SR (2010). Neural pathogenesis of enterovirus 71 infection. Microbes Infect.

[28] Wang C, Zhou R, Zhang Z, Jin Y, Cardona CJ, Xing Z (2015). Intrinsic apoptosis and proinflammatory cytokines regulated in human astrocytes infected with enterovirus 71. J Gen Virol.

[29] Yang CH, Liang CT, Jiang ST, Chen KH, Yang CC, Cheng ML, et al. A novel murine model expressing a chimeric mSCARB2/hSCARB2 receptor is highly susceptible to oral infection with clinical isolates of enterovirus 71. J Virol. 2019;93:e00183–19. 10.1128/JVI.00183-19 PMC6532076 30894476

[30] Yu P, Gao Z, Zong Y, Bao L, Xu L, Deng W (2015). Distribution of enterovirus 71 RNA in inflammatory cells infiltrating different tissues in fatal cases of hand, foot, and mouth disease. Arch Virol.

[31] Huang HI, Lin JY, Chen HH, Yeh SB, Kuo RL, Weng KF (2014). Enterovirus 71 infects brain-derived neural progenitor cells. Virology.

