# Supplementary material for: Mechanism for the lethal effect of enterovirus A71 intracerebral injection in neonatal mice
Source: Lab Invest. 2019 Dec 19;100(4):596–605. doi: 10.1038/s41374-019-0351-5 (PMC7096333; doi:10.1038/s41374-019-0351-5)
Supplement: Supplementary file 1 — Supplemental Figure [file 41374_2019_351_MOESM1_ESM.pdf]

Supplementary

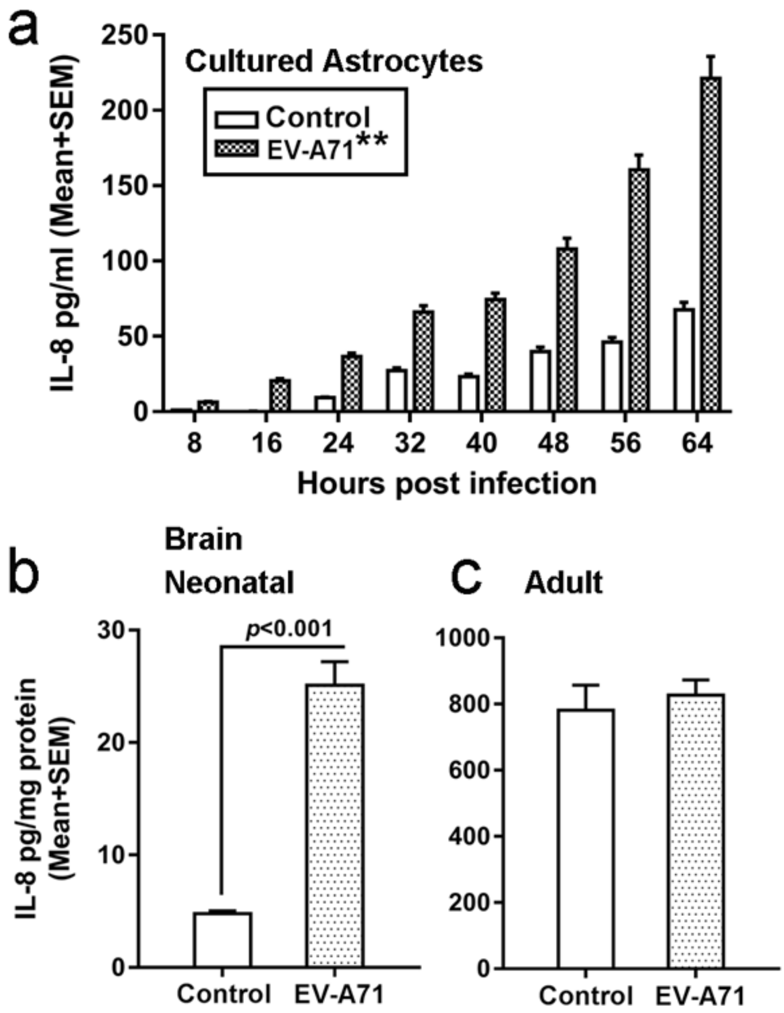

S1. Detection of IL-8 in astrocytes *in vitro* and in the brains of mice with EV-A71 infection *in vivo*. IL-8 released by astrocytes from 8 to 64 hours post EV-A71 infection (a). IL-8 detected in brainstem homogenates from neonatal (b) and adult (c) mice 4 days post infection. $^{**}p \leq 0.01$ in comparison with the corresponding control group.
